# *FOXG1* Dose in Brain Development

**DOI:** 10.3389/fped.2019.00482

**Published:** 2019-11-22

**Authors:** Nuwan C. Hettige, Carl Ernst

**Affiliations:** ^1^Department of Human Genetics, McGill University, Montreal, QC, Canada; ^2^Psychiatric Genetics Group, Douglas Mental Health University Institute, Montreal, QC, Canada; ^3^Department of Psychiatry, McGill University, Montreal, QC, Canada; ^4^Integrated Program in Neuroscience, McGill University, Montreal, QC, Canada

**Keywords:** *FOXG1*, BF-1, iPSCs, neurodevelopment, neural stem cell, gene dosage

## Abstract

Brain development is a highly regulated process that involves the precise spatio-temporal activation of cell signaling cues. Transcription factors play an integral role in this process by relaying information from external signaling cues to the genome. The transcription factor Forkhead box G1 (*FOXG1*) is expressed in the developing nervous system with a critical role in forebrain development. Altered dosage of *FOXG1* due to deletions, duplications, or functional gain- or loss-of-function mutations, leads to a complex array of cellular effects with important consequences for human disease including neurodevelopmental disorders. Here, we review studies in multiple species and cell models where *FOXG1* dose is altered. We argue against a linear, symmetrical relationship between *FOXG1* dosage states, although *FOXG1* levels at the right time and place need to be carefully regulated. Neurodevelopmental disease states caused by mutations in *FOXG1* may therefore be regulated through different mechanisms.

## Introduction

Mammalian brain development involves cell proliferation and differentiation of cells into specific types, usually in response to diffusible signaling cues and cell-cell interactions. It is the precise spatio-temporal order of cell division, growth, motility, and cell fate determination that leads to the specified structures of the mammalian central nervous system, including the forebrain (telencephalon), midbrain (mesencephalon), and hindbrain (rhombencephalon) (1, 2). External signals may initiate specific cell programs but inside each cell is a complex messenger system whereby critical signals for development are relayed to the genome to induce gene expression and to make mRNA and protein for specific functions. Transcription factors play a critical role in this process, forming an output for external signaling cues and second messengers by directly interacting with the genome. Forkhead Box G1 [*FOXG1*; previously known as *BF-1* (3)] is one such factor and is necessary for the development of the telencephalon (4, 5), though is also expressed in the retina, inner ear, and olfactory bulb. The homozygous loss of *Foxg1* in mouse leads to a severe reduction in telencephalic structures (5), and the loss of one copy of *FOXG1* in human leads to postnatal microcephaly, severe developmental delay, and structural brain deficits such as cerebral atrophy, gyral simplification, hypomyelination, and a thin or absent corpus callosum (6).

The concept of gene dose refers to the amount of product (mRNA and/or protein) produced from a given allele or mRNA. In some cases, allelic expression is imbalanced where one allele may contribute more product than another allele (7). This can be a normal state and does not necessarily imply disease, as evidenced by imprinting effects and monoallelic expression from several genes (7, 8). In other cases, a change in gene dose (through deletion or duplication, for example) may have no effect on proliferation or cell fate determination. Why is it that some regions of the genome are dosage sensitive while others are not (9)? In the case of Down Syndrome (DS), for example, all genes on chromosome 21 are increased by 50% (three alleles per gene instead of two), yet not all genes show increased expression (10), nor is it the case that genes that show increased expression contribute to the DS phenotype. It is thought that increased dose from only a few genes [the DS critical region (11)] are required for the disease phenotype. This implies that increased expression from several genes on chromosome 21 have no effect on cell fate and so these genes are presumably not dosage sensitive.

**Figure 1:**
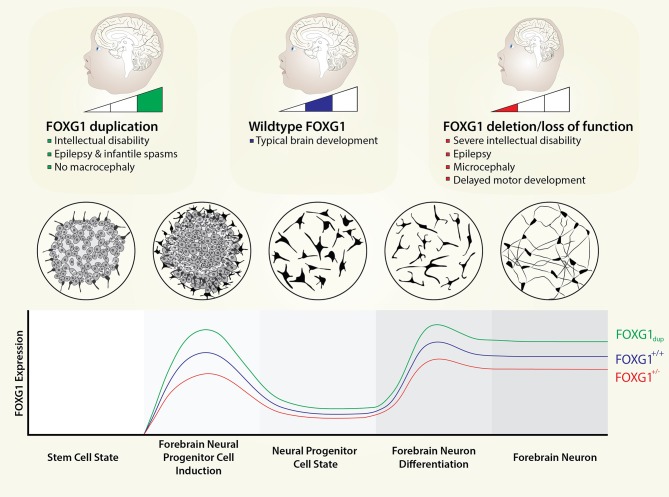
*FOXG1* dosage in neurodevelopment. *FOXG1* expression levels are currently unknown; however, *FOXG1* is one of the earliest genes to be expressed in mammalian telencephalon. We envision *FOXG1* expression being activated well before terminal differentiation of forebrain neurons and even forebrain neural progenitors. Here, we depict expression patterns from a stem cell state to forebrain neurons, with induction and maintenance states defined. Circular plates show the appearance of cells *in vitro* for the different cell states. We model *FOXG1* dosage changes as linear with respect to gene dosage, even though the molecular effects of FOXG1 protein (e.g., interaction with different proteins or different genomic regions) are not necessarily linear with respect to protein dose.

There are a few known biological reasons why dosage can be important for some genes and not for others. Often, it is intimately related to the biological activity of the encoded protein. For example, some transcription factors may need to partner with another factor to exert an effect and in the absence of one transcription factor the other will bind with a different partner, leading to different cellular effects. The chromatin remodeling complexes BAF (SWI/SNF) and TIP60 are a good example of this (12–14), where several proteins associate to drive an effect, but the same complex with a few changes in binding patterns can lead to a different cellular effect. Genes that code for these specific proteins would thus be considered dosage sensitive. Several human syndromes that cause neurodevelopmental disease can be considered sensitive to gene dose (15); including *FOXG1* deletion syndrome, though it is not clear why *FOXG1* dosage is so critical (Figure 1). To address this question, we have laid out this review by looking at *FOXG1* dose in multiple model systems, diseases, and tissue types, and analyzing molecular interacting patterns and signaling pathways that could contribute to dosage sensitivity. Our hope is that integrating information from different research areas and studies might better illuminate the role of *FOXG1* dose in human neurodevelopment.

## Forkhead Box Family

Forkhead box (Fox) transcription factors belong to a superfamily of related proteins characterized by a winged-helix DNA-binding domain approximately 110 residues long (16, 17). Fox transcription factors bind a similar DNA sequence, albeit with different affinities, due to their highly conserved DNA-binding motif. These genes have been ubiquitously present during the evolutionary history of vertebrates and invertebrates, from worms to humans (18–23). The evolutionary expansion of Fox gene family members has been driven by the increased developmental and tissue complexity required of higher organisms (24).

Fox protein regulation and function vary significantly between families, arising in part from sequence variation outside of the DNA-binding forkhead domain, allowing for differential regulation and functional diversification. As a result, Fox proteins have been found to participate in numerous physiological processes and biological functions including embryonic development and organogenesis, cell cycle regulation, metabolism control, stem cell niche maintenance, and signal transduction (24, 25). While their role in developmental patterning is well known, many Fox genes continue to be expressed in post-embryonic structures, suggesting there are other important functions that have yet to be elucidated (26).

The total number of Fox genes varies widely among different organisms. *C. elegans* have 15 compared to 44 known Fox genes in humans (24). The mammalian forkhead family of transcription factors are categorized into subclasses A to S based on sequence similarity within and outside of the forkhead box (25, 27). The divergent sequences outside of the conserved DNA-binding domain likely distinguish between the function of these proteins, in addition to their distinct temporal and spatial expression patterns.

## Forkhead Box G1 (*FOXG1*)

Like all Fox family members, the winged-helix transcription factor forkhead box G1 (*FOXG1;* formerly named forebrain-restricted transcription factor BF-1, qin, Chicken Brain Factor 1, or XBF-1) is characterized by unique sequences of amino acids within the forkhead-binding domain (FHD) (25). *FOXG1* is expressed in a variety of nervous system cell types and tissues, including the cerebral cortex, telencephalon, inner ear, retina, olfactory epithelial cells, and other neural and sensory tissues in mammals (28). The timing of its expression also varies by tissue type.

In humans, *FOXG1* is located on chromosome 14q12 and contains only one coding exon (29, 30). The amino acid sequence from the FHD to C-terminal domain is highly conserved (29), with the N-terminal domain being more variable among species. In addition to the FHD, *FOXG1* consists of a 10-residue histone demethylase (*KDM5B*; previously *JARID1B*)-binding domain (JBD) and a 20-residue Groucho (Gro)-binding domain (GBD). The FHD consists of three alpha helices and one beta hairpin (two beta strands and one loop) (6).

*FoxG1* primarily acts as a transcriptional repressor in the embryonic telencephalon (31, 32). From multiple studies of *FoxG1* deficiency in animal models, it has become apparent that *FoxG1* plays a vital role in brain development, ranging from telencephalon specification and patterning and neuronal differentiation, to maintenance and survival of mature neurons (26). Mouse knockout (KO) studies of *Foxg1* revealed it to be a regulator of neurogenesis in which it regulates early cortical cell fate by coordinating the expression of an early transcriptional network in the cerebral cortex (33–35). Thus, *FoxG1* is not just one of many important transcription factors in brain development; rather it is considered a pioneer transcription factor in that it is one of the earliest expressed in this cell type and can alter the structure of chromatin to allow other factors to bind (36).

## Role of *FOXG1* in the Development of the Vertebrate Telencephalon

The transcription factor *Foxg1* is essential for the normal development of the telencephalon. The vertebrate forebrain (prosencephalon) arises from the largest portion of the neural tube—a structure derived from the neuroectoderm composed of a layer of neuroepithelial cells. From there, bilateral swellings known as telencephalic vesicles are generated in the most rostral region to form the telencephalon (37). The cerebral cortex forms from the dorsal telencephalon, while the basal ganglia develop from the ventral telencephalon. These dorsal and ventral regions are patterned by the activities of many secreted morphogens produced by different signaling centers (38–43).

Multiple signals are required for the correct specification of the telencephalon including bone morphogenetic proteins (*BMPs*), wingless/integrated proteins (*WNTs*), extracellular signal fibroblast growth factor 8 (*FGF8*), and sonic hedgehog (*SHH*) (44–47). In the embryonic telencephalon, *SHH* is produced ventrally, *FGF8* is produced rostrally and multiple *BMPs* and *WNT* proteins are produced caudo-medially (35). These morphogens and others coordinate the expression of transcription factors including *FOXG1* that regulate subsequent telencephalic development. The fine-tuning of *Foxg1* expression levels by specific spatio-temporal signals from other morphogens and their second messenger relays is what allows for the precise development of the telencephalon; though exactly how this occurs is not well understood. *Foxg1* is one of the earliest transcription factors to be expressed during early neurogenesis and responds to a variety of signaling cues (48). A detailed description of *Foxg1* activity during brain development is reviewed in Kumamoto and Hanashima and Danesin and Houart (26, 49). Here, we will briefly summarize the spatio-temporal patterning of mammalian brain development at the onset of *Foxg1* expression, using mice as a model system.

At embryonic day 8.5 (E8.5), *Foxg1* expression is present in the most rostral region of the neural tube. *Foxg1* and *Shh* both promote *Fgf8* expression in the anterior neural ridge (ANR) to pattern the nascent telencephalon (40). The ANR is a region in the neural plate which acts as a secondary organizer and secretes signaling molecules that generate the anterior-posterior patterning of the forebrain. *Foxg1* directly promotes *Fgf8* expression, while *Shh* indirectly promotes *Fgf8* expression by inhibiting *Gli3* repression of *Fgf8* (40). As a result, *Shh* allows the formation of a ventral telencephalic subdivision by inhibiting the dorsalizing effects of *Gli3*. Both *Foxg1* and *Fgf8* are required to form the complete telencephalon.

By embryonic day 9 (E9), *Foxg1* expression is contained in the telencephalic neuroepithelium, including the progenitor cells of the cerebral cortex, the basal ganglia and the olfactory bulb (50). At E9.5, *Foxg1* expression declines in the dorsomedial telencephalon and the dorsal midline, though *Foxg1* expression persists in the ventral telencephalon. At E12.5, *Foxg1* is expressed in telencephalic neural progenitors and absent from the rest of the neural tube. Lastly, according to coronal sections of 4-month old mice brains, *Foxg1* expression remains restricted to cells derived from the telencephalic neuroepithelium, including the cerebral cortex and the hippocampus.

## *FOXG1* Syndrome

*FOXG1* syndrome is a rare neurodevelopmental disorder characterized by abnormal brain development and function due to mutations in one copy of *FOXG1*.

*FOXG1* syndrome (OMIM #613454) was first thought to be a congenital variant of Rett Syndrome (RTT) with many overlapping features of typical RTT but with differences in disease onset and symptoms (51). The features of RTT generally include a rapid regression in language and motor skills between the ages of 6–18 months in which affected individuals demonstrate repetitive and stereotypic hand movements, severe intellectual disability (ID), and social impairment. Since the original description of RTT in 1966 (52) and its characterization in 1983 (53), a RTT diagnosis was based only on consensus clinical criteria until mutations in *MECP2* were identified in almost all classical RTT cases (54). As a result, RTT patients were characterized as having typical RTT if they fit the consensus criteria or atypical RTT if they had the congenital form (51, 55).

The *FOXG1* gene was first implicated in the congenital form of RTT in 2005, when a 7-year old girl with a 720-kb inversion in chromosome 14q12 disrupting *FOXG1* was identified (56). The affected girl displayed severe ID, tetraplegia, and structural brain abnormalities including microcephaly, myelination defects, and agenesis of the corpus callosum. Soon afterwards, clinical reports of children with facial dysmorphisms, microcephaly, and ID were identified with 14q12 interstitial deletions overlapping *FOXG1* (57, 58). Other reports of atypical RTT associated *FOXG1* as the causal gene following the discovery of interstitial 14q12 *de novo* deletions in patients with no observable *MECP2* mutations (59–61). From these findings, *FOXG1* was recognized as a strong candidate gene for the syndrome, due to its high expression in the developing brain and the reported developmental abnormalities in the telencephalon of both heterozygous and homozygous mouse mutants (62, 63). Since then, retrospective molecular screenings for *FOXG1* mutations were done in large cohorts of typical and atypical RTT patients (64–66). These screens of RTT patients with no mutations in *MECP2* later identified non-sense, frameshift, and missense mutations in *FOXG1*.

## *FOXG1* Dose and Cell Survival

In the developing brain, neural stem cells (NSCs) are controlled by a tightly regulated series of signals that coordinate proliferation and differentiation into different neural cell types (neurons, astrocytes, and oligodendrocytes) that ultimately populate the mature brain (67). NSCs are defined as self-renewing, multipotent cells that generate neurons, astrocytes, and oligodendrocytes. Neural progenitor cells (NPCs) have a limited life span, less self-renewal capacity, and may be multipotent or unipotent (68, 69). Subventricular zone NSCs first divide symmetrically to expand the population of ventricular zone progenitor cells then switch to divide asymmetrically (67). Asymmetric cell division gives rise to a progenitor cell and radial glia or neurons which migrate and form the cortical layers.

NSCs must continually counterbalance pro-death and pro-survival signals to ensure the appropriate numbers of cells in the progenitor pool and the developing cortex (70–73). Mediators of these processes can either increase or decrease cell-death signals or increase or decrease pro-survival signals. Cell death involves a strictly regulated series of events and is an essential aspect of an organism's life. The controlled nature of the initiation, execution, and termination of the cell death process is commonly referred to as apoptosis (71). Apoptosis is a series of specific biochemical and morphological changes that lead to the degradation of cells and their contents in a controlled manner. The distinguishable morphological features of apoptosis include chromatin condensation, nuclear fragmentation, cytoplasmic condensation, membrane blebbing, and nucleus, and inter-nucleosomal cleavage of DNA (74–76). Toward the end of the process, the apoptotic cell is converted into membrane-bound fragments called apoptotic bodies which are quickly eliminated via phagocytosis (77, 78). The caspases are major mediators of this process in that they perform the controlled demolition of cell components (79).

The tight regulation of NSC apoptosis will have a dramatic effect on the final size of the NSC pool (72). This should be distinguished from the more widely studied form of cell or synaptic pruning of projection neurons during neurodevelopment. At the early stages of embryogenesis, large-scale apoptosis occurs in the brain, eliminating a majority of the newly generated neuronal population following neurogenesis (70). This is also demonstrated in the proliferative regions of adult brain, the subventricular zone (SVZ) and dentate gyrus (DG) (80–82).

How might dosage of *FOXG1* affect control NSC apoptosis? Caspases exist in the cell as inactive procaspase monomers that need to dimerize to be active, and do this in response to signaling cues (83). Active caspase assembly involves specific adapter proteins, and the amount of dimerized active caspases results in a positive feedback loop to activate other caspases (79). Given that the total number of active caspases determines outcome and caspases can be regulated at the procaspase and dimerization level, one could imagine a situation where *FOXG1* either regulates the expression of a negative regulator of these factors or that *FOXG1* protein can interact and inhibit caspase dimerization. There are no studies to our knowledge on direct or indirect effects of *FOXG1* on caspase regulation. However, this model provides an example of how dose could lead to dramatic effects on apoptosis.

### Complete Loss of *Foxg1*

Mice with a homozygous loss of *Foxg1* display severe abnormalities in telencephalon development and die shortly after birth (5, 84). In particular, the telencephalon from *Foxg1* null mice are significantly smaller than normal from E10.5 to perinatal death (6). The *Foxg1* null telencephalon is also enriched for dorsal markers while ventral cell fates are not (5, 33, 40, 50, 85, 86). Dorsal telencephalic neuroepithelial cells also differentiate prematurely, leading to the early depletion of neural progenitors. These results suggest that *Foxg1* controls the morphogenesis of the telencephalon by regulating the rate of neuroepithelial cell proliferation and the timing of neuronal differentiation (5, 84). One study examined the outcome of homozygous loss-of-function *Foxg1* models by making a DNA binding defective version of the gene called BF1^NHAA^ (86). The authors suggest that this led to reduced proliferation and precocious differentiation of *Foxg1*-deficient neural progenitors (86). Conditional deletion of *Foxg1* from pyramidal neurons (selective deletion using CRE/LoxP system driven by *NeuroD*) (87) showed that in *Foxg1*-cKO (conditional KO) the cortex was substantially thinner, the ventricles were enlarged, and the intermediate zone was not well-defined at postnatal day 0 (P0). Lastly, the corpus callosum was missing throughout the anterior-posterior axis, and the hippocampus failed to develop in *Foxg1*-cKO mice (87). The authors suggest an important signaling complex for projection neurons that may be important in corpus callosum formation including *Znf513, Slit3, Reelin*, and *Robo1*.

Other models of complete loss of *Foxg1* have also been investigated. In a homozygous knockout neuronal cell line, embryoid bodies (EBs) derived from induced pluripotent stem cells (iPSCs) were significantly smaller (88), supporting a role for *Foxg1* in cell survival. *In vivo* adult neurogenesis models support this finding. (89) conditionally ablated *Foxg1* to create homozygous knockouts specifically in the dentate gyrus. They used a tamoxifen inducible, Frizzled9 Cre/LoxP approach for this and show almost complete loss of subgranular zone cells. Apoptosis occurred as early as half a day following *Foxg1* deletion with cell death persisting until at least P7. There was a significant decrease in the number of postmitotic neurons at P14 which was attributed to increased cell death following postnatal *Foxg1* ablation rather than impaired neurogenesis in the DG.

Complete loss of *Foxg1* can be considered an extreme version of a loss of gene dosage, though in cases of complete loss it is difficult to argue that gene dosage matters (there are several syndromes in human that require complete loss of a gene product, e.g., some recessive disorders, and where loss of one allele has no effect). For this, we require an investigation into models with increased dose of *FoxG1* and reduced, but not absent *FoxG1*.

### Increased Dose of *FoxG1* and Cell Survival

#### FoxG1 Over-expression in Chick and Xenopus

In cranial neural tube slices of White Leghorn chick embryos, Ahlgren et al. (85) performed retroviral gene transfer to overexpress the avian homolog of *FoxG1, V-qin*, in the telencephalon and to ectopically express it in the mesencephalon, rhombencephalon, and spinal cord (85). The ectopic expression of *FoxG1* resulted in a selective overgrowth of the telencephalon and mesencephalon (midbrain) but not in more posterior brain regions. As well, there was a marked thickening of the neuroepithelium. Interestingly, a separate experiment demonstrated that retroviral expression of *FoxG1*^*NHR*−*AAA*^ (virus containing the *FoxG1* construct with the DNA binding domain inactivated) resulted in no observable phenotype. This finding suggested that the brain overgrowth is mediated through the DNA-binding domain of *FoxG1* (85). Ahlgren et al. (85) concluded that the observed overgrowth was not due to an increase in proliferation rates (85). Embryos examined 2–3 days after retroviral infection demonstrated no significant increase in BrdU incorporation in the neural tube. Similarly, there was no detectable effect of *FoxG1*^*NHR*−*AAA*^ on proliferation as measured by BrdU or mitotic index (85). Rather than uncontrolled proliferation, Ahlgren et al. suggested that the absence of normal programmed cell death was associated with the brain overgrowth observed in *FoxG1* overexpressing chicks (85). The authors used DAPI as an indicator of apoptotic nuclei and observed that dying cells appeared small and bright. Cellular counts revealed a significant decrease in the number of apoptotic nuclei in the anterior neural tube including both the telencephalon and mesencephalon. Furthermore, control retroviruses and *FoxG1*^*NHR*−*AAA*^ did not yield a significant change in the apoptotic index compared to embryos with no virus infection.

In *Xenopus laevis* embryos in which *FoxG1* (known as *XBF-1*) is overexpressed, studies revealed an expansion of the telencephalic progenitor population (90, 91). According to Hardcastle and Papalopulu, embryos injected with a high *XBF-1* concentration show increased proliferation over an area of expanded or ectopic neuroectoderm, such that the normally bilayered neuroectoderm becomes multilayered (91). *XBF-1* injected embryos also demonstrated proliferating neural precursor cells in lateral domains where post-mitotic cells would normally be found. Overall, the authors show that a high dose of *XBF-1* causes tissue outgrowths in the ectoderm, increases proliferation, and inhibits the expression of the cyclin-dependent kinase (cdk) inhibitor p27XIC (91). Bourguignon et al. demonstrated that neuronal differentiation is specifically suppressed in cells in which *XBF-1* is expressed at high levels (90). This was seen also via the thickening of the ectoderm in developing *Xenopus* models as well as through an increase in the number of proliferating progenitor cells in place of differentiated neurons in the anterior neural plate (90). These studies support a model whereby increased *XBF-1* leads to more proliferation of precursor cells possibly through suppression of neuron differentiation.

#### Duplication of FOXG1 in Human Populations

“Natural” experiments exist in humans whereby mutations have arisen on chromosome 14q12 where *FOXG1* is duplicated leading to three gene copies instead of two. Pontrelli and others recently reviewed 15 cases with duplications on chromosome 14q12 all of which included *FOXG1* (92), where epilepsy and cognitive impairment with dysmorphic features are the common phenotypes of this cohort. There was no identifiable microcephaly, though there is also no macrocephaly, arguing against a simple balanced model of *FOXG1* to drive proliferation, at least in human. Some amount of *FOXG1* may be required to ensure enough progenitor cells are made. Too much *FOXG1* however, may not affect this specific process which may involve an interaction with specific proteins that govern the generation of progenitor cells. The epilepsy and intellectual disability phenotypes in the duplication cases may arise from completely different mechanisms than from loss of *FOXG1* dosage, i.e., the interaction of *FOXG1* with different molecules or with different genomic regions.

#### Increase of FOXG1 in Human Tumors and Neurodevelopmental Disease Associated With Macrocephaly

Cancers are broadly defined as a group of diseases that involve abnormal cell growth with the potential to invade or spread to other parts of the body. Tumors are large masses that are often the result of this abnormal growth of cells. Resistance to cell death is an important feature of cancers, where apoptosis has been established as a mechanism of anti-cancer defense. Gliomas are a common form of brain cancer characterized by excessive cell proliferation and aggressive infiltration (93). Notably, *FOXG1* has been shown to be upregulated in glioma as well as ovarian cancer and medulloblastoma (94–96), and to have important driver effects. To examine the role of *FOXG1* in glioma, Chen et al. examined *FOXG1* expression in two cultured glioma cell lines (U87MG and SHG44) and found elevated *FOXG1* expression in U87MG cells (93). A lentivirus-mediated expression system was used to overexpress *FOXG1* in SHG44 cells and a lentivirus-mediated shRNA was used to knock down *FOXG1* in U87MG. The results of these expression studies demonstrated that cell proliferation was decreased as a function of downregulated *FOXG1*. Similarly, increased cell proliferation was associated with increased *FOXG1* expression. The authors further questioned whether the change in the proliferation rate was attributed to altered apoptotic activity. In *FOXG1*-overexpressing SHG44 cells, apoptosis appeared to be reduced given by the decreased expression of caspase-9, 8 and 3 and the cleaved versions of these pro-apoptotic proteins. Furthermore, expression of these caspases was elevated in the *FOXG1* knockdown U87MG cells, indicating increased apoptotic activity. Together, these results suggest that *FOXG1* has a pro-survival function and that expression is negatively correlated with glioma cell apoptosis (93).

Given the purported pro-proliferation and anti-differentiation activity of *FOXG1*, a study by Wang et al. (97) hypothesized that *FOXG1* expression supported the resistance of glioblastoma multiforme (GBM) cells against temozolomide (TMZ) treatment. TMZ is a DNA methylation agent and drug resistance-modifying agent that induces G2/M arrest and apoptosis. Upon TMZ treatment, viability of GBM cells was assessed using an MTT assay (apoptotic assay) which demonstrated significantly reduced cell viability—defined as the ratio of initial cell number minus dead cell number to the initial cell number (97). GBM cells transiently overexpressing *FOXG1* in combination with TMZ treatment showed significantly improved cell viability, indicating that *FOXG1* resisted the anti-proliferation ability of TMZ treatment (97).

In a smaller study, Adesina et al. (98) demonstrated that *FOXG1* is significantly differentially overexpressed in aggressive medulloblastoma subtypes from four publicly available gene expression profiling data sets. As a result, the authors attempted to examine the genome-wide effect of down-regulating *FOXG1* expression in DAOY (a medulloblastoma cell line) by running an mRNA expression profile of 44,000 genes using the sh*FOXG1*, shLuciferase, and the UT DAOY cell lines. Whole-genome expression analyses revealed pathways affected by decreased *FOXG1* including those involved in cell adhesion and migration (98). As expected, changes in expression were seen in genes previously implicated in cancer. There also appeared to be a variety of altered genes involved in cell survival or anti-apoptotic activity (98). In a separate experiment, the authors demonstrated that mice xenografts injected with DAOY cells demonstrated enhanced survival when transfected with sh*FOXG1* knockdown constructs as opposed to sh-Luciferase. Overall, these studies offer evidence for the overexpression of *FOXG1* in mediating excessive cell survival in glioma and medulloblastoma, respectively (93, 98).

Idiopathic autism spectrum disorder refers to individuals where no underlying cause for the disorder has been identified. (99) suggest that *FOXG1* may act as a convergence point for these ASDs associated with macrocephaly and modeled these patients in human stem cells. Gene expression profiling of neurons derived from different patient lines revealed that overexpression of *FOXG1* was ubiquitous in their transcriptomic profiles. The authors also observed an excitatory/inhibitory neuron imbalance in brain organoids generated from proband iPSCs, such that *FOXG1* may be partially involved in the overproduction GABAergic neurons.

Studies from model organisms, human duplication cases, neurodevelopmental disorders with macrocephaly and no mutation in *FOXG1*, and human tumors suggest that cell survival and tissue growth are sensitive to *FOXG1* gene dosage. Tipping the balance of *FOXG1* toward overexpression leads to a reduction in cell death and tissue overgrowth. Brain overgrowth and tumor formations are logical consequences of *FoxG1* overexpression as its role in promoting proliferation and cell survival are amplified beyond normal levels. This idea needs to be tempered with the results from the human duplication cases where no brain overgrowth was observed (92), arguing against a simple *FOXG1* dosage model. While the data do support a *FOXG1* dosage sensitive model in brain cells, it may be that the mechanism important in reduced dosage of *FOXG1* operates on different molecules than those that are important where there is too much *FOXG1*, something we call an asymmetric dosage sensitivity model.

### Heterozygous Models of *FOXG1* Syndrome

#### Foxg1^+/−^ Mouse Models

*Foxg1* heterozygous mice were first generated while making *Foxg1* homozygous KO mice and were considered as controls (i.e., before the human heterozygous deletion syndrome was identified, highlighting the importance of heterozygotes). This was attributed to the fact that several initial studies reported that mice with a single allele of *Foxg1* develop an apparently normal cerebral cortex (5, 33, 50, 86, 100). *Foxg1* heterozygous mice did not exhibit the severe cortical defects in patterning observed in the null mice (100), at least on cursory observation. Closer investigation of *Foxg1*^+/−^ mice identified smaller cortical volumes and *Foxg1* heterozygous mice showed a reduction in layer II/III thickness associated with microcephaly and impaired hippocampal neurogenesis (62, 101). The *Foxg1*^+/−^ model also showed hyperlocomotion, impaired habituation in the open field and a severe deficit in contextual fear conditioning (62, 63, 101). The cerebral cortex, hippocampus and striatum were observed to have reduced volumes in the *Foxg1*^+/−^ mice (62, 63), though this may be strain or genetic background dependent. For example, the forebrain of heterozygous *Foxg1* mice maintained on the C57BL/6J background had severely impaired development. However, *Foxg1*^+/−^ mice of the *Foxg1-tet* line and *Foxg1-lacZ* and *Foxg1-cre* mice maintained on a mixed background, did not display reduced cortical thickness. This suggests that reduced but not absent *Foxg1* in mice displays complex interactions with brain development.

#### Heterozygous Loss of FOXG1 in Humans

Clinical data on several *FOXG1* deletion syndrome patients have been reviewed and discussed in this review; however, understanding why a loss or mutation in one copy of *FOXG1* leads to microcephaly and severe intellectual disability in humans is unknown. Human-derived iPSCs now make it feasible to generate isogenic, patient-derived neurons to investigate neurodevelopment and to perform functional genetic studies (102). Patriarchi et al. generated iPSC-derived neurons from *FOXG1*^+/−^ patients and suggested that there is an imbalance in excitatory/inhibitory (E/I) synaptic protein expression in patient neurons compared to controls (103). However, these data do not explore the dynamics of *FOXG1* dose as neurons develop. It seems reasonable to suspect that the molecular mechanism of disease will arise early on as cells differentiate and any overt cellular phenotype at a mature cell stage is a passenger effect to an earlier problem in cell differentiation. It is these early molecular mechanisms that need to be assessed to understand how *FOXG1* dose leads to a reproducible, robust cellular phenotype. To this end, a recent study was able to generate human stem cells where *FOXG1* dose could be fine-tuned (104). Studies such as these will become important in titrating specific doses at specific times for *in vitro* neurodevelopment.

### Binding Partners That May Mediate *FOXG1* Dosage Effects

*FOXG1* dose appears to be critical for the proper differentiation or proliferation of specific cell types. One way that protein levels (dose) can exert its effects is by binding to other molecules. Dose effects can be revealed by the need to compete with other proteins to interact with a given protein or protein complex. The reduced amount of *FOXG1* may allow a protein complex to perform different functions, whereas too much may allow *FOXG1* to outcompete other proteins for binding sites where it has lower affinity.

#### Groucho (Gro)/Transducin-Like Enhancer of Split (TLE)

*FoxG1* is known to interact directly with Groucho (*Gro*)/Transducin-like enhancer of split-1 (*TLE1*) by forming a transcription repression complex with co-repressors of the *TLE* family (105, 106). *TLE* family members are transcriptional repressors that lack a DNA binding motif and so are dependent on other factors for this function, like *Foxg1*. Among mammals, there are four full-length *TLE* family members (*TLE1-4*) and two shorter isoforms–Groucho-related gene product (Grg) 5 and 6. Only full-length *TLE* and Grg6 proteins contain a conserved C-terminal WD40 repeat domain mediating interaction with *FoxG1*. Grg6 acts as a dominant-negative regulator of *FoxG1*:TLE transcriptional repressor complexes (107). Grg6 interferes with the binding of *TLE1* to *FoxG1* and does not repress transcription when targeted to DNA. Moreover, co-expression of *Grg6* and *FoxG1* in cortical progenitor cells leads to a decrease in the number of proliferating cells and increased neuronal differentiation (107). Furthermore, Roth et al. show that *Xenopus tropicalis TLE2 (*a closely related family member to *TLE1)* physically interacts with *FoxG1* in the ventral telencephalon (subpallium) (108) via a conserved N-terminal Engrailed Homology 1 (EH1) motif. Knocking down *TLE2* leads to impaired development of the ventral telencephalon, similar to the knockdown of *FoxG1*. This suggests that *TLE2* is a spatially restricted member of the Groucho/TLE family, which interacts with *FoxG1* to specify and promote the development of the ventral telencephalon. The dynamic interplay of TLE and Grg proteins shows just how dynamic altering the total level of *Foxg1* protein could be. The binding affinities of each would be critical to determining outcome, and suggest that a simple linear model (more expression of *FOXG1* = more binding with TLE) is not necessarily correct.

#### Lysine Demethylase 5B (KDM5B)

*FOXG1* cooperates with KDM5B (previously JARID1B or PLU-1), a histone demethylase, to potentially regulate cell proliferation and differentiation. The interaction between KDM5B and *FOXG1* is mediated by a conserved interacting motif (Ala-*X*-Ala-Ala-*X*-Val-Pro-*X*_4_-Val-Pro-*X*_8_-Pro; termed the VP motif) in both proteins (109). The interaction between *FOXG1* and the transcriptional repressor KDM5B is of functional importance for early brain development (110). In particular, during mouse embryogenesis, *KDM5B* expression overlaps with *FOXG1* expression both spatially and temporally (111). While the two interact directly, KDM5B also acts as a repressor of *FOXG1* expression (112). KDM5B then can both regulate the expression of *FOXG1* and bind to *FOXG1* protein, possibly forming important regulatory loops. Dosage change in *FOXG1* would thus have important consequences on the activities of KDM5B.

KDM5B is an H3K4 demethylase (mono-, di-, and tri-), and therefore has a role in removing an important mark of actively transcribed regions. KDM5B is classified as a repressive chromatin writer and so loss of function would lead to a more permissive (i.e., more gene expression) chromatin state. KDM5B is predominantly expressed during embryonic development, including embryonic stem cells (ESCs) and also the adult brain, testis, eye, spleen and thymus (111–113). It has also been identified as an oncogene in many cancer types (114). This suggests that while it is essential for normal development, any perturbation to gene expression may induce abnormal phenotypes related to growth and survival.

#### Polycomb Complex Protein BMI-1 (BMI-1)

*FOXG1* and BMI-1 expression levels are tightly correlated with each other in a close expression loop to affect neural progenitor cell survival (115). *BMI-1* is part of the polycomb repressive complex 1, a transcriptional repressor complex known to interact with multiple proteins (116). PRC1 is thought to repress gene expression by affecting the level of histone H2A variants in nucleosomes (117), levels of which determine the stem-like state of a cell. While *FOXG1* and BMI-1 may not physically interact, their tight regulatory relationship might suggest that the proper dose of *FOXG1* is important for PRC1-mediated gene repression in nerve cells, perhaps as a proper guide to the correct genomic coordinates.

### Signaling Mechanisms that May Contribute to or Be Affected by *FoxG1* Dosage

#### FGF8 Signaling

FGF8 is thought to directly affect *FOXG1* during neurodevelopment to specify and pattern the ventral telencephalon (40, 84, 118). FGF8 is a morphogen meaning it derives its function from amount or concentration along a specific gradient. Morphogens are an appealing model to explain why *FOXG1* dose may be important since more or less *FOXG1* might lead to more or less FGF8, or vice versa. At neural plate stages, *Fgf8* induces and/or maintains *Foxg1* expression in the anterior neural ridge (119). *Foxg1* then restricts the expression of *Bmp4* to the midline where BMP4 is believed to induce apoptosis. Analysis of serial sections of forebrains from normal, *Fgf8* function eliminated, and *Fgf8* function reduced animals confirmed that the *Foxg1* and *Bmp4* expression domains in the midline were complementary. These observations support the hypothesis that FGF8 regulates telencephalic cell survival in part via a *Foxg1* pathway and that either eliminating or increasing *Fgf8* expression decreases *Foxg1* pathway activity; whereby reducing *Fgf8* expression increases it (119).

#### PI3K-Akt Signaling

The PI3K (phosphatidylinositide-3′-OH kinase)-Akt pathway (120) promotes neuronal survival by inactivating the cell death machinery and repressing pro-apoptotic gene expression (121, 122), likely through IGF-1 (123). This signaling cascade activates CK1 and AKT, both of which can target *FOXG1* (122). *Foxg1* may be imported into the nucleus of cells through its phosphorylation by CKI which promotes NPC differentiation into neurons, while *Foxg1* phosphorylation by Akt at Thr271 leads to *Foxg1* nuclear export. Loss of *FOXG1* dose may mean there are less *FOXG1* targets available to be phosphorylated and that a critical mass of *FOXG1* may need to be phosphorylated for AKT to execute its cellular programming.

#### TGF-β Signaling

The transforming growth factor-β (TGF-β) pathway consists of multiple cytokines that control a wide variety of biological activities including apoptosis, cell proliferation, differentiation, cell adhesion, and embryonic development through TGF-β and other receptors and Smad transducer proteins (124, 125). Studies have shown that *FoxG1* may act as a negative regulator of TGF-β signaling pathway by binding to the MH2 of Smads -1, -2, -3, and -4 (96, 126). This association blocks the binding of Smad proteins to DNA and results in the inhibition of TGF-β signaling (127). *FoxG1* has been shown to inhibit expression of the cyclin-dependent kinase (CDK) inhibitor *p21*^*WAF*1/*CIP*1^, which is normally transcriptionally activated by TGF-β signaling, in glioblastoma and the neuroepithelium (96, 128). While TGF-β signaling is complex and context dependent, some of the effects of decreased *FOXG1* dose could be exerted through its interactions with the SMAD proteins.

## Conclusion

Altering *FOXG1* dose leads to severe consequences in different cell types though this may be through asymmetric mechanisms. In this review, we have shown the results across different systems of increased or decreased *FOXG1* dosage, as well as the different binding partners or signaling systems that may explain why dosage is important. These data support a non-linear model whereby *FOXG1* interactions with different players may be governed by substrate affinity or phosphorylation states, arguing against any simplistic model of *FOXG1* dose. How and when *FOXG1* is expressed, how *FOXG1* is stabilized or degraded, and background genetics will all be important determinants of the effects of *FOXG1* gain or loss on brain development.

## Author Contributions

NH and CE both evaluated the scientific literature and wrote the review together.

### Conflict of Interest

The authors declare that the research was conducted in the absence of any commercial or financial relationships that could be construed as a potential conflict of interest.
